# Efficacy of intensive antibiotic regimens on postcraniotomy fever and cerebrospinal fluid examination results in patients with infratentorial surgeries

**DOI:** 10.1097/MD.0000000000032214

**Published:** 2022-12-16

**Authors:** Yuan Yao, Xian Wang

**Affiliations:** a Department of Neurosurgery, The First Affiliated Hospital of Yangtze University, Jingzhou, China; b Department of Pharmacy, The First Affiliated Hospital of Yangtze University, Jingzhou, China.

**Keywords:** acoustic neuroma, infratentorial surgeries, postcraniotomy fever, trigeminal neuralgia

## Abstract

Postcraniotomy fever is a common complication in patients undergoing infratentorial surgeries. There are few studies about it and the efficacy of intensive antibiotic regimens, which remain to be studied. We carried out a retrospective study in patients undergoing infratentorial surgeries to analyze the factors associated with postcraniotomy fever and further investigated the efficacy of different antibiotic regimens on fever and abnormal cerebrospinal fluid (CSF) results. Among the 115 patients, 74 (64.3%) had fever after craniotomy. Univariate analysis results showed that disease type, drainage tube placement, duration of drainage tube, and intraoperative bleeding volume were associated with postcraniotomy fever in patients undergoing infratentorial surgeries (*P* < .05). The multivariate analysis results showed that the duration of drainage tube (odds ratio = 1.010, 95% confidence interval: 1.232–6.178, *P* = .014) and duration of surgery (odds ratio = 1.010, 95% confidence interval: 1.002–1.017, *P* = .013) were associated with postcraniotomy fever in these patients. After treatment with different antibiotic regimens, the changes of white blood cells, sugar, chlorine and protein in CSF in patients with intensive antibiotic regimens were −638.48 × 10^6^/L, 0.25 mmol/L, −0.76 mmol/L and −0.16 g/L respectively, which did not show significant differences when compared with ordinary antibiotic regimens (*P* > .05). Most cases of fever at the early stage after craniotomy in patients with infratentorial surgeries were nonintracranial infectious. Intensive antibiotic regimens did not show obvious advantages in improving the body temperature and CSF examination results. It is not necessary to use intensive antibiotic regimens too early, such as carbapenems or linezolids.

## 1. Introduction

Fever after surgery is a common complication, including early postsurgery fever and later postoperative fever.^[1,2]^ The incidence of postoperative fever in different surgeries varies. The reported incidence of postoperative fever after cardiac surgeries was 43%, after endoscopic endonasal surgeries was 17%, after cervical fusion surgeries was 25%, and after retrograde internal surgeries was 17.5%.^[3–6]^ Fever is also a common complication of craniotomy procedures. After craniotomy procedures, the reported incidences of fever were 8.9% to 81.7%.^[7,8]^ Especially, among patients undergoing infratentorial surgeries, we empirically observed postcraniotomy fever in many cases at an early stage. These patients often received intensive antibiotic regimens, such as carbapenems or linezolid, but the majority of cerebrospinal fluid (CSF) bacterial cultures were negative. In addition, the operation time in cases of infratentorial surgeries is often longer. Whether postcraniotomy fever is associated with the operation time and whether intensive antibiotic regimens can improve the fever and CSF examination results in these cases are the primary purposes of this retrospective study.

## 2. Materials and Methods

### 2.1. Study design and ethical statement

We retrospectively analyzed the clinical characteristics and CSF examination results of patients who underwent infratentorial surgeries between January 2019 and June 2022. Data were collected through the electronic medical record system (EMRS) at the First Affiliated Hospital of Yangtze University with the consent of the ethics committee. The study was conducted according to the Strengthening the Reporting of Observational Studies in Epidemiology guidelines. The study was approved by the Institutional Review Board of the First Affiliated Hospital of Yangtze University.

### 2.2. Data source and study population

The hospital’s EMRS was used to identify patients who met the following eligibility criteria: The inclusion criteria were as follows: 18 years or older; diagnosis of posterior fossa diseases, including acoustic neuroma, cerebellar tumor, trigeminal neuralgia, facial spasm, cerebellar infarction, and cerebellar hemorrhage confirmed by computed tomography findings and clinical manifestations; and had undergone craniotomy procedures to treat the posterior fossa diseases. The exclusion criteria were as follows: fever before craniotomy, rebleeding at the operation site, infections far away from the operation site, miscellaneous causes and drug-associated fever, and combined other tumors. In total, 115 eligible patients were included.

The criteria for cases with fever were as follows: had a temperature ≥37.5°C, going along with the criteria in China and in some references^[9–11]^; and satisfied the above inclusion criteria. The criteria for cases without fever were as follows: had a temperature <37.5°C, and satisfied the above inclusion criteria. If a patient was defined as having a fever, we made a diagnosis of bacterial meningitis according to CSF leukocytes, CSF protein, CSF glucose, and bacterial culture results.^[12]^

**Figure 1.**
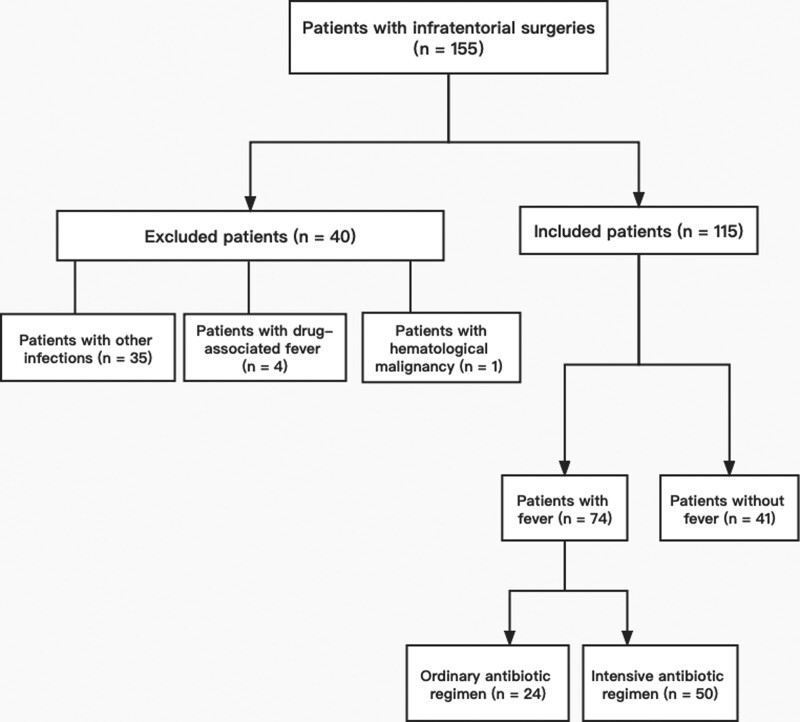
Flow chart of the study.

The criteria for cases with intensive antibiotic regimens were as follows: received antibiotics including meropenem, linezolid, tegacyclin, or vancomycin; and satisfied the criteria for fever. The criteria for cases with ordinary antibiotic regimens were as follows: received antibiotics including cephalosporins or clindamycin and satisfied the criteria for fever. The flow chart of the study was shown in Figure 1.

### 2.3. Collected information

Dr Wang retrieved data from the medical records of eligible patients in the EMRS and recorded them in the data collection tables. Dr Yao checked the accuracy of the data. The following data were collected: temperature, sex, age, disease type, combined diseases, preoperative blood cell counts, CSF examination results, surgery duration, intraoperative bleeding volume, drainage tube duration, hospital stay duration, and hospitalization expenses. The occurrence of postcraniotomy fever was assessed based on the recorded body temperatures. Fever was defined as a temperature >37.5ºC. The dates when the temperature initially exceeded 37.5ºC after surgery and when the temperature returned to the normal level were also recorded.

CSF samples were obtained by lumbar puncture from patients with fever. All patients with fever required at least 1 lumbar puncture to acquire CSF examinations, including white blood cells (WBCs), protein, sugar, and chlorine by 2 examiners. Bacterial cultures of CSF samples were also performed.

The primary endpoints were postcraniotomy fever and efficacy of intensive antibiotic regimens on changes of CSF examination results including WBCs, sugar, chlorine, and protein.

### 2.4. Statistical analysis

SPSS software (version 23.0, IBM SPSS Inc., Chicago) was used for the data analysis. Quantitative data are described as “mean ± standard deviation” or “median ± interquartile range” according to the distribution. If the distribution was normal and the variance was homogeneous, an independent sample t-test was used for the analysis. If the distribution was normal but the variance was not homogeneous, a corrected t-test was used. If the normal distribution could not be satisfied, the Mann–Whitney *U* test was used. Qualitative data were described by “n, %” and compared using the Pearson chi-square (χ^2^) test, continuity correction, or Fisher exact tests. Multivariate logistic regression was used to analyze the factors associated with postcraniotomy fever in patients undergoing infratentorial surgeries. All *P*-values were 2-sided and statistical significance was set at *P* < .05.

## 3. Results

### 3.1. Demographics and characteristics

Of 115 patients, 60 (52.2%) were women, and 55 (47.8%) were men. The mean age of the patients was 58 years. There were 34 (29.6%) patients with posterior cranial fossa tumors, 24 (20.9%) with hemifacial spasm, 35 (30.4%) with trigeminal neuralgia, 20 (17.4%) with cerebellar hemorrhage, and 2 (1.7%) with cerebellar infarction. Among the 115 patients, 3 (2.6%) had diabetes, 45 (39.1%) had hypertension, and 28 (24.3%) had cerebral infarction. Among all included patients undergoing craniotomy, there were 39 cases undergoing craniectomy, including 28 cases in patients with fever and 11 cases in patients without fever. However, there was no significant difference between the 2 groups (*P* = .232). All patients received prophylactic antibiotic after surgery. Clindamycin was used in 9 cases including 6 cases in patients with fever and 3 cases in patients without fever. The first-generation cephalosporins (cefradine or ceftazole) were used in 4 cases including 2 cases in patients with fever and 2 cases in patients without fever. The second-generation cephalosporins (cefmendo/cefmetazole) were used in 87 cases including 52 cases in patients with fever and 35 cases in patients without fever. The third-generation cephalosporins (cceftazidime/cephalosporin oxime) were used in 15 cases including 14 cases in patients with fever and 1 case in patients without fever. There was also no significant difference between the 2 groups (*P* = .079).

### 3.2. Postcraniotomy fever

Among the 115 included patients, 41 (35.7%) had no fever, and 74 (64.3%) had fever. The average temperatures in patients with and without fever were 38.3ºC and 36.6 ºC respectively. The median time when fever occurred was 2 days after surgery, and the average duration of fever was 4 days in patients with fever. In terms of accompanying symptoms, there were 48 (64.9%) patients with headache and 10 (13.5%) with signs of meningeal stimulation in patients with fever. The duration of hospital stay (23 days) and hospitalization expenses (53830.9 Renminbi) increased significantly in patients with fever (*P* < .001) (Table 1).

**Table 1:** The univariate analysis results of postcraniotomy fever in patients with infratentorial surgeries.

Variable	Cases with fever (n = 74)	Cases without fever (n = 41)	*P* value
Age (yr)	57 ± 15	63 ± 12	.083
Sex (n, %)			
Male	39 (52.7)	16 (39.0)	.160
Female	35 (47.3)	25 (61.0)	
Disease types (n, %)			.001
Posterior cranial fossa tumors	26 (35.1)	8 (19.5)	
Hemifacial spasm	8 (10.8)	16 (39.0)	
Trigeminal neuralgia	24 (32.4)	11 (26.8)	
Cerebellar hemorrhage	16 (21.6)	4 (9.8)	
Cerebellar infarction	0	2 (4.9)	
Prophylactic antibiotic after surgery			.079
Clindamycin	6 (8.1)	3 (7.3)	
First-generation cephalosporins	2 (2.7)	2 (4.9)	
Second-generation cephalosporins	52 (70.3)	35 (85.4)	
Third-generation cephalosporins	14 (18.9)	1 (2.4)	
Preoperative blood cell counts			
WBCs (×10^9^/L)	5.6 ± 4.4	5.3 ± 2.0	.432
RBCs (×10^12^/L)	4.3 ± 0.5	4.2 ± 0.5	.269
Hemoglobin (g/L)	134.0 ± 23.5	130.8 ± 23.1	.470
Combined disease (n, %)			
Diabetes mellitus	1 (1.4)	2 (4.9)	.289
Hypertension	27 (36.5)	18 (43.9)	.435
Cerebral infarction	16 (21.6)	12 (29.3)	.360
Placement of drainage tube (n, %)	43 (58.1)	14 (34.1)	.014
Duration of drainage tube (d)	3 ± 2	2 ± 0	.004
Duration of surgery (min)	203 ± 162	180 ± 110	.059
Craniectomy	28 (37.8)	11 (26.8)	.232
Intraoperative bleeding volume (mL)	300 ± 275	100 ± 150	<.001
Temperature (ºC)	38.3 ± 0.8	36.6 ± 0.6	<.001
Duration of hospital stay (d)	23 ± 10	18 ± 8	<.001
Hospitalization expenses (RMB)	53,830.9 ± 37,081.4	37,623.2 ± 17,571.1	<.001

RBCs = red blood cells, RMB = renminbi, WBCs = white blood cells.

### 3.3. Risk factors of postcraniotomy fever

There were no significant differences in age, sex, combined diseases, hemoglobin, red blood cell and WBC counts in blood before surgery between patients with and without fever (*P* > .05) (Table 1). Among the 74 patients with fever, 26 (35.1%) had posterior cranial fossa tumors, 8 (10.8%) had facial spasm, 24 (32.4%) had trigeminal neuralgia, and 16 (21.6%) had cerebellar hemorrhage. Among the 41 patients without fever, 8 (19.5%) had posterior cranial fossa tumors, 16 (39%) had facial spasms, 11 (26.8%) had trigeminal neuralgia, 4 (9.8%) had cerebellar hemorrhage, and 2 (4.9%) had cerebellar infarction. Among the patients with fever, the proportion of posterior cranial fossa tumors patients is the highest; among the patients without fever, the proportion of hemifacial spasm patients is the highest.

Drainage tubes were placed in 43 of the 74 patients with fever and in 14 of the 41 patients without fever. The median duration of drainage was 3 days in patients with fever and 2 days in patients without fever. The average duration of surgery in patients with fever was 203 minutes, in patients without fever was 180 minutes. The median intraoperative bleeding volumes in patients with and without fever were 300 mL and 100 ml respectively.

Twenty-six posterior cranial fossa tumors cases in patients with fever and 16 facial spasms cases in patients without fever were further analyzed. Drainage tubes were placed in 26 posterior cranial fossa tumors cases, but not in 16 facial spasms cases. The duration of surgery and intraoperative bleeding volume in these cases were also analyzed. The duration of surgery in posterior cranial fossa tumors cases was 350.12 ± 101.11 minutes, significantly longer than in facial spasm cases (204.69 ± 60.76 minutes), *P* < .001. The intraoperative bleeding volume in posterior cranial fossa tumors cases was 325 ± 100 mL, significantly more than in facial spasm cases (100 + 37.5 mL), *P* < .001.

Univariate analysis results showed that disease type (*P* = .001), placement of drainage tube (*P* = .014), duration of drainage tube (*P* = .004) and intraoperative bleeding volume (*P* < .001) were associated with fever (Table 1). The multivariate analysis results showed that the duration of drainage tube (odds ratio = 2.759, 95% confidence interval: 1.232–6.178, *P* = .014) and duration of surgery (odds ratio = 2.759, 95% confidence interval: 1.002–1.017, *P* = .013) were associated with fever after craniotomy in patients with infratentorial surgeries (Table S1, Supplemental Digital Content, http://links.lww.com/MD/I81).

### 3.4. Efficacy of different antibiotic regimens on fever and CSF examination results

Among 24 patients with ordinary antibiotic regimens, there were 10 cases treated with cefotaxime, 9 cases with ceftazidime, 3 cases with cefmendor, 1 case with cefotaxime sulbactam, and 1 case with clindamycin. Among 50 patients with intensive antibiotic regimens, there were 26 cases treated with meropenem alone, 19 cases with meropenem and linezolid, 2 cases with linezolid and cephalosporins, 1 case with meropenem and tegacyclin, 1 case with meropenem and vancomycin, and 1 case with meropenem, tegacyclin and linezolid. After treatment with different antibiotics, the median duration of fever in patients with intensive antibiotic regimens was 6 days, which was significantly longer than that in patients with ordinary antibiotic regimens (3 days) (*P* < .001).

**Figure 2.**
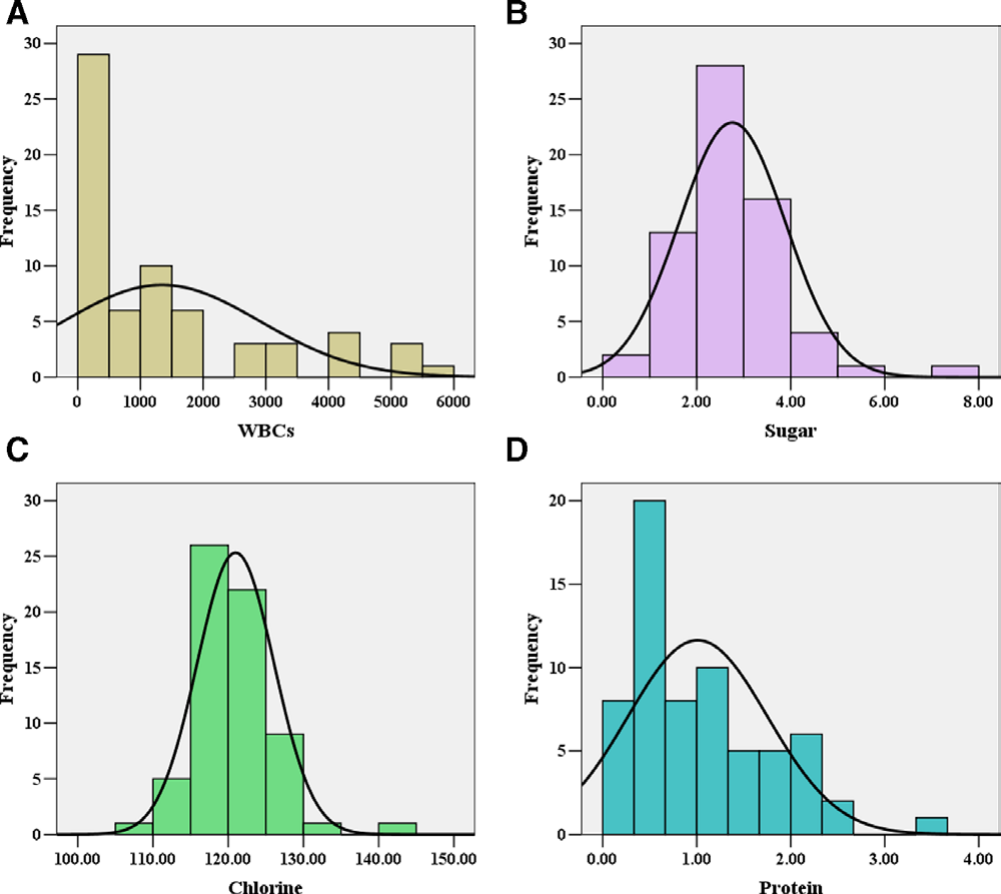
Distribution of first cerebrospinal fluid examination results in patients with fever. (A) WBCs: (860 ± 1831) × 10^6^/L; (B) sugar: (2.76 ± 1.13) mmol/L; (C) chlorine: (120.98 ± 5.12) mmol/L; (D) protein: (1.01 ± 0.74) g/L. WBCs = white blood cells.

Nine patients were unable to obtain CSF results by lumbar puncture because of lumbar spinal stenosis or rejection of this operation. Among the 65 febrile patients undergoing lumbar puncture, the median WBC count in the first CSF examination after fever was 860 × 10^6^/L. The median levels of sugar and chlorine were 2.76 mmol/L and 120.98 mmol/L respectively. The median level of protein in first examined CSF was 1.01 g/L, including IgG (101.63 mg/L) and IgM (17.20 mg/L) (Fig. 2). Finally, only 2 (3.1%) patients had positive bacterial culture results from CSF. Human staphylococcus was found in 1 patient and saprophytic staphylococcus was found in the other patient. Both bacteria were methicillin sensitive staphylococcus, and the 2 patients were treated with meropenem.

**Figure 3.**
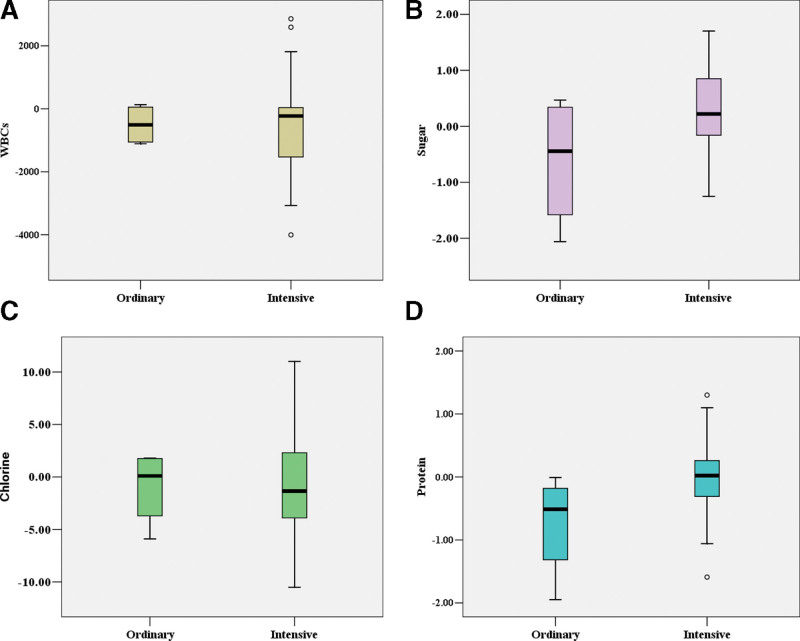
Efficacy of different antibiotic regimens on CSF examination results in patients with fever. The differences of changes in WBCs (A), sugar (B), chlorine (C), and protein (D) in the CSF between patients with intensive antibiotic regimens and ordinary antibiotic regimens were not significant (*P* > .05). CSF = cerebrospinal fluid, WBCs = white blood cells.

Figure 3 showed the efficacy of different antibiotic regimens for fever and CSF examination results in patients with fever. After treatment with different antibiotic regimens, the changes of WBC counts in CSF among patients with intensive and ordinary antibiotic regimens were −638.48 × 10^6^/L and −521.80 × 10^6^/L, respectively (*P* = .865). The changes of sugar in CSF were 0.25 mmol/L and −0.52 mmol/L respectively (*P* = .082). The changes of chlorine in CSF were −0.76 mmol/L and −0.52 mmol/L respectively (*P* = .915). The changes of protein in CSF were −0.16 g/L and −0.59 g/L (*P* = .272). Changes in the levels of IgG and IgM (*P* = .393) also did not show significant differences. The specific values of the changes were listed in Table 2.

**Table 2:** Efficacy of different antibiotic regimens on fever and cerebrospinal fluid examination results in patients with fever.

	Intensive antibiotic regimens (n = 50)	Ordinary antibiotic regimens (n = 24)	*P* value
Sugar (mmol/L)	0.25 ± 0.87	−0.52 ± 1.05	.082
Chlorine (mmol/L)	−0.76 ± 4.87	−0.52 ± 3.30	.915
Protein (g/L)	−0.16 ± 0.79	−0.59 ± 0.81	.272
WBCs (×10^6^/L)	−638.48 ± 1491.67	−521.80 ± 559.77	.865
IgG (mg/L)	3.65 ± 65.10	−26.15 ± 273.25	.393
IgM (mg/L)	−0.45 ± 9.85	0.20 ± 20.83	.393

WBCs = white blood cells.

## 4. Discussion

There were 74 cases of fever among 115 patients undergoing infratentorial surgeries, and the incidence of fever was 64.3%, higher than that reported in a study on supratentorial tumor surgeries and the study by Sanchez et al.^[13,14]^ The difference in the incidence between the 2 studies could be attributed to the difference in the included patients. In contrast to the study by Sanchez et al, we included patients with trigeminal neuralgia, facial spasm, cerebellar hemorrhage, and cerebellar infarction.

A retrospective study carried out in pediatric patients with craniotomy reported that 43% of the patients developed fever 12 days after craniotomy.^[15]^ In the study conducted by de Almeida AN,^[16]^ all included patients had fever within 4 days after craniotomy. Wang et al showed that fever occurred 1 to 5 days after craniotomy in patients with posterior fossa tumors.^[17]^ Consistent with this study, the median time in our study when fever occurred was 2 days after craniotomy, and the median duration of fever was 4 days. Therefore, fever after craniotomy in patients with infratentorial surgeries possibly occurred early and lasted for a short time. In our study, fever was accompanied by headaches in 64.9% of patients, with signs of meningeal stimulation in 13.5% of patients.

Some studies have explored the correlation between clinical characteristics and postcraniotomy fever. In a study conducted by de Almeida,^[16]^ the size of the excised hemisphere was possibly associated with the temperature level. Phung et al reported that intraoperative bleeding volume, placement and duration of the drainage tube were possibly associated with fever after craniotomy.^[15]^ In the study carried out by Gillow SJ, the percentage of noninfective fever after intracerebral hemorrhage was 71%. The amount of intracranial hemorrhage, intraventricular hemorrhage, and placement of a drainage tube were possibly associated with fever.^[18]^ However, there are few studies on the risk factors of fever after infratentorial surgeries. Our study confirmed the association between the duration of the drainage tube and fever after craniotomy in patients undergoing infratentorial surgeries. The disease types had significant impact on the incidence of postoperative fever. In our study, the proportion of posterior fossa tumors cases in patients with fever is the highest, and the proportion of hemifacial spasm cases in patients without fever is the highest. We found that drainage tubes were placed in 26 tumor cases. Through further analysis, we also found significantly more intraoperative bleeding volume and significantly longer duration of surgery. We speculated that intraoperative bleeding volume and duration of surgery might be the cause of the disease type affecting the patient’s fever.

Among 74 patients with fever, increases of WBCs and protein in the CSF were observed; however, the levels of sugar and chlorine were very close to normal. Sajjad et al conducted a 14-year investigation of complications after craniotomy. The results showed that there was only 1 case of postcraniotomy infection among 305 patients undergoing glioma surgeries, and 8 cases of postcraniotomy infections among 120 patients undergoing meningioma surgeries. The incidence of postcraniotomy fever caused by infection was 2.1%.^[19]^ The incidences of meningitis and bacterial meningitis were 4.54% and 0.87% respectively in 1146 patients with tumors in the cerebellopontine angle region. Significantly elevated WBC counts and relatively low level of glucose in CSF were confirmed in patients with bacterial meningitis, while slightly elevated WBC counts and normal level of sugar in CSF were observed in patients with chemical meningitis.^[14]^ Consistent with these 2 studies, fever after craniotomy in our study was mostly noninfectious. A positive bacterial culture in the CSF was demonstrated in only 2 patients (3.1%). Local effusion of the surgical wound, damaged necrotic tissue in the operation area, limitation of the operation space in the posterior cranial fossa, proximity to the thermoregulatory center, and posterior fossa muscle exudates were possible causes of fever after infratentorial surgeries.^[16,17,20]^

After fever onset, the majority of patients were treated with intensive antibiotics. Some studies have focused only on the efficacy of prophylactic antibiotics for fever or meningitis after craniotomy. Prophylactic antibiotics reduced the incidence of incision infection after craniotomy from 8.8% to 4.6% and reduced the incidence of bacterial meningitis from 1.63% to 1.50%.^[21]^ One meta-analysis including 2365 patients supported the efficacy of prophylactic antibiotics in reducing the incidence of meningitis after craniotomy.^[22]^ There were very few studies on the efficacy of different antibiotic regimens on postcraniotomy fever and CSF examination results in infratentorial surgeries. In our study, we compared the efficacy of intensive and ordinary antibiotic regimens on CSF examination results after craniotomy in patients with infratentorial surgeries. We did not find significantly decreased counts of WBCs in the CSF of patients receiving intensive antibiotic regimens. Moreover, intensive antibiotic regimens did not show obvious advantages in improving glucose, chlorine, and protein levels in CSF.

We found possible factors affecting postcraniotomy fever in patients undergoing infratentorial surgeries and the efficacy of different antibiotic regimens on fever and CSF examination results. However, our study had some limitations. The CSF examination results in patients with fever were partially missing and deleted, which could have led to a bias. In addition, our sample size is small. This was a preliminary study, and we will expand the sample size for the next step.

Through our preliminary study, we believed that most cases of fever at the early stage after craniotomy in patients with infratentorial surgeries were nonintracranial infectious. Intensive antibiotic regimens did not show obvious advantages in improving the body temperature and CSF examination results. It is not necessary to use intensive antibiotic regimens too early, such as carbapenems or linezolids. We will carry out a large-sample study of fever after craniotomy in patients undergoing infratentorial surgeries.

## Author contributions

**Conceptualization:** Yuan Yao, Xian Wang.

**Data curation:** Xian Wang.

**Formal analysis:** Xian Wang.

**Methodology:** Yuan Yao.

**Project administration:** Yuan Yao.

**Writing – original draft:** Yuan Yao.

**Writing – review & editing:** Xian Wang.

## Supplementary Material
