# Supplementary material for: Efficacy of intensive antibiotic regimens on postcraniotomy fever and cerebrospinal fluid examination results in patients with infratentorial surgeries
Source: Medicine (Baltimore). 2022 Dec 16;101(50):e32214. doi: 10.1097/MD.0000000000032214 (PMC9771276; doi:10.1097/MD.0000000000032214)
Supplement: Supplementary file 1 [file medi-101-e32214-s001.pdf]

Supplementary Table1

The multi-variate analysis results to assess risk-factors of postcraniotomy fever

							95.0% CI for EXP(B)	
	**B**	**S.E.**	**Wald**	**df**	**Sig.**	**Exp(B)**	**Lower**	**Upper**
Duration of drainage tube	1.015	0.411	6.086	1	.014	2.759	1.232	6.178
Duration of surgery	0.009	0.004	6.164	1	.013	1.010	1.002	1.017
Constant	-3.949	1.563	6.382	1	.012	0.019		
